# Supplementary material for: Diagnostic yield of exome sequencing in nonobstructive azoospermia (NOA): A systematic review and meta-analysis
Source: PLoS One. 2025 Dec 16;20(12):e0338892. doi: 10.1371/journal.pone.0338892 (PMC12707621; doi:10.1371/journal.pone.0338892)
Supplement: S1 Text — (DOCX) [file pone.0338892.s001.docx]

Supplementary File S1

**Search histories**

**Ovid MEDLINE(R) ALL <1946 to March 07, 2025>**

1 azoosperm*.mp. or Azoospermia/

2 (ASPERMIA or oligospermia).mp.

3 (Nonobstructive azoospermia or NOA).mp.

4 azoospermic.mp.

5 (male infertility or spermatogenesis or spermatogenic defect*).ti,ab,kw.

6 Infertility, Male/

7 1 or 2 or 3 or 4 or 5 or 6

8 Exome Sequencing/

9 exome sequenc*.ti,ab,kw.

10 WES.tw.

11 Exome/

12 DNA test*.mp.

13 Molecular Diagnostic Techniques/ or molecular test*.mp.

14 8 or 9 or 10 or 11 or 12 or 13

15 7 and 14

**Embase 1947-Present, updated daily**

1 azoosperm*.mp. or Azoospermia/

2 (ASPERMIA or oligospermia).mp.

3 (Nonobstructive azoospermia or NOA).mp.

4 azoospermic.mp.

5 (male infertility or spermatogenesis or spermatogenic defect*).ti,ab,kw.

6 male infertility/

7 1 or 2 or 3 or 4 or 5 or 6

8 Whole Exome Sequencing/

9 exome sequenc*.ti,ab,kw.

10 WES.tw.

11 Exome/

12 DNA test*.mp.

13 molecular diagnosis/ or molecular test*.mp.

14 8 or 9 or 10 or 11 or 12 or 13

15 7 and 14

16 limit 15 to "remove medline records"

**Interface - EBSCOhost Research Databases**

**Database - CINAHL**

| **#** | **Query** |
| --- | --- |
| S3 | S1 AND S2 |
| S2 | TX ( exome sequenc* or WES ) OR TX molecular diagnos* |
| S1 | TX (Azoospermia or ASPERMIA or oligospermia or Nonobstructive azoospermia or NOA) |

**Scopus (Elsevier)**

TITLE-ABS-KEY ( azoospermia OR aspermia OR oligospermia OR nonobstructive AND azoospermia ) ) AND ( TITLE-ABS-KEY ( exome AND sequenc* OR wes OR molecular AND diagnos* ) ) AND ( LIMIT-TO ( EXACTKEYWORD , "Human" ) OR LIMIT-TO ( EXACTKEYWORD , "Male" ) )

**Cochrane Central Register of Controlled Trials**

**Issue 2 of 12, February 2025**

#1 ( exome sequenc* or WES OR molecular diagnos* )

#2 azoospermia or ASPERMIA or oligospermia or Nonobstructive azoospermia

#3 #1 and #2

**Science Citation index-expanded (Web of Science)**

Azoospermia or ASPERMIA or oligospermia (Topic) AND Exome Sequencing or WES or molecular diagnos* or molecular test* (Topic)
