# Supplementary material for: Diagnostic yield of exome sequencing in nonobstructive azoospermia (NOA): A systematic review and meta-analysis
Source: PLoS One. 2025 Dec 16;20(12):e0338892. doi: 10.1371/journal.pone.0338892 (PMC12707621; doi:10.1371/journal.pone.0338892)
Supplement: S2 Text — (DOCX) [file pone.0338892.s002.docx]

**Supplementary File 2. GRADE evidence profile for each pooled analysis assessing diagnostic yield of ES in NOA**

| **Quality assessment** | |  |  |  |  |  | **Summary of findings** | | |  |
| --- | --- | --- | --- | --- | --- | --- | --- | --- | --- | --- |
| **No of Studies (Design)** | **Limitation** | **Inconsistency** | **Indirectness** | **Imprecision** | **Publication bias** |  | **Number of patients** | | **Proportion (95%CI)** |  |
|  |  |  |  |  |  |  | **Events** | **Total** |  | **Certainty** |
| Nine  Observational [15-23] | No Serious | Serious^a^  I^2^=90% | No Serious | No Serious | Undetected |  | 270 | 1,728 | 0.15 [0.10; 0.20] | Low |
| Eight  Oservational [15-19, 21-23] | No Serious | Serious^a^  I^2^=79% | No Serious | No Serious | Detected |  | 92 | 804 | 0.14 [0.08; 0.19] | Very low |
| **Sensitivity analysis** |  |  |  |  |  |  |  |  |  |  |
| Eight  Observational [16-23] | No Serious | Serious^a^  I^2^=66% | No Serious | No Serious | Undetected |  | 256 | 1,437 | 0.16 [0.12; 0.20] | Low |
| Eight  Observational [15,17-23] | No Serious | Serious^a^  I^2^=90% | No Serious | No Serious | Undetected |  | 262 | 1,705 | 0.14 [0.09; 0.18] | Low |
| Eight  Observational [15-16,18-23] | No Serious | Serious^a^  I^2^=91% | No Serious | No Serious | Undetected |  | 267 | 1,693 | 0.15 [0.10; 0.21] | Low |
| Eight  Observational [15-17,19-23] | No Serious | Serious^a^  I^2^=91% | No Serious | No Serious | Undetected |  | 262 | 1,673 | 0.15 [0.09; 0.20] | Low |
| Eight  Observational [15-18,20-23] | No Serious | Serious^a^  I^2^=90% | No Serious | No Serious | Undetected |  | 248 | 1,632 | 0.13 [0.08; 0.18] | Low |
| Eight  Observational [15-20,22-23] | No Serious | Serious^a^  I^2^=91% | No Serious | No Serious | Undetected |  | 267 | 1,715 | 0.14 [0.09; 0.19] | Low |
| Eight  Observational [15-21,23] | No Serious | Serious^a^  I^2^=91% | No Serious | No Serious | Undetected |  | 250 | 1,543 | 0.15 [0.10; 0.21] | Low |
| Eight  Observational [15-22] | No Serious | Serious^a^  I^2^=91% | No Serious | No Serious | Undetected |  | 256 | 1,622 | 0.15 [0.09; 0.21] | Low |

Note: GRADE=Grading of Recommendations, Assessment, Development, and Evaluation.

a. I^2^ value >50%

Low quality: Our confidence in the effect estimate is limited: the true effect may be substantially different from the estimate of the effect.

Very low quality: We have very little confidence in the effect estimate: the true effect is likely to be substantially different from the estimate of effect.
