# Supplementary material for: Diagnostic yield of exome sequencing in nonobstructive azoospermia (NOA): A systematic review and meta-analysis
Source: PLoS One. 2025 Dec 16;20(12):e0338892. doi: 10.1371/journal.pone.0338892 (PMC12707621; doi:10.1371/journal.pone.0338892)
Supplement: Attachment — Submitted filename: reviewer notices.docx [file pone.0338892.s006.docx]

**Introduction:**

- Procedural Details: For instance, micro-TESE techniques and IVF-ICSI protocols are important, but might be trimmed in favor of staying focused on the genetic narrative.

-Redundant Phrasing: Some information is repeated (e.g., the diagnostic limitations of current methods); a tighter structure can avoid diluting your key message.

-Spermatogenesis Stages: Briefly mention the complexity without breaking down every sub-stage unless it's directly relevant to gene-pathology links.

-I'd appreciate even just 3–5 well-established genes associated with NOA (e.g., TEX11, NR5A1, SYCP3, DMRT1)

**Results**

ome minor points:

“Publication bias were…” → should be “was.”

“TEST outcomes” likely meant “TESE outcomes.”

“Include study” should be “included study.”

Main Concern:

-The diagnostic yield of 15% with high heterogeneity (I² = 90%) weakens the generalizability of the findings. Even after excluding the outlier study, the I² drops to 79%, which still reflects substantial variability.

Recommendation: Clarify sources of heterogeneity—e.g., were there differences in ES platforms, inclusion criteria, or variant interpretation pipelines? A subgroup or meta-regression analysis would strengthen your conclusions.

-The initial funnel plot bias is statistically non-significant (P=0.46), which is good—but after exclusion, bias becomes significant (P=0.01). That’s a red flag.

Suggestion: Provide a rationale for why publication bias increased after refining your dataset. Was the excluded study larger/smaller or methodologically distinct?

-Mentioning 264 genes across 270 cases underscores the extreme genetic heterogeneity of NOA. However, listing only the top eight without describing their functions or known pathogenicity misses an opportunity.

Stronger framing would include:

1.Whether these gene variants were classified as pathogenic/likely pathogenic or VUS.

2.Functional categories (e.g., meiosis-related, DNA repair)

3.Recurrence rates—were any genes found in independent cohorts?

The phenotype-genotype analysis is a great addition. Highlighting positive sperm retrieval rates by histopathological subtype adds clinical relevance.

Still, the n is very low for retrieval outcomes (only 45 cases with TESE info). Drawing broad predictive conclusions from this is risky.

Recommendation: Be more cautious in your language; frame these associations as preliminary observations rather than definitive predictors.

**Discussion**

1. Structure and Flow

The discussion would benefit from clearer paragraph divisions. Right now, multiple ideas are packed into long blocks of text. Suggest splitting into sections like:

-Diagnostic yield and heterogeneity

-Genetic insights and gene-specific findings

-Clinical correlation with TESE outcomes

-Broader implications and future directions

2. Overuse of Numbers Without Interpretation

While numbers like “264 genes in 270 cases” or “2,274 testis-expressed genes” are impressive, they need context. What proportion are known monogenic causes? How many are novel?

3. Insufficient Functional Context for Key Genes

AR, TEX11, FANCM, etc., are cited as “top genes,” but their biological roles and clinical significance are not explained. Including 1–2 lines describing their mechanistic relevance would add depth.

4. TESE Outcomes: Careful Framing

The 11.11% TESE success rate in ES-positive cases is a striking finding, but be cautious in interpretation:

Small sample size (only 45 reported cases)

Possibly skewed by selection bias

Could reflect enrichment for more severe phenotypes

Frame it as hypothesis-generating, not definitive.

5. AI Model Mention

The mention of an AI model is promising but abrupt. If it's important, integrate it earlier with more detail or drop it for cohesion.
