# Supplementary material for: Diagnostic yield of exome sequencing in nonobstructive azoospermia (NOA): A systematic review and meta-analysis
Source: PLoS One. 2025 Dec 16;20(12):e0338892. doi: 10.1371/journal.pone.0338892 (PMC12707621; doi:10.1371/journal.pone.0338892)
Supplement: Attachment — Submitted filename: reviewer comments.docx [file pone.0338892.s008.docx]

**Reviewer #1: The research is very important, but need some modification to be more relevant to the aim of the study.**

**Introduction**

- Procedural Details: For instance, micro-TESE techniques and IVF-ICSI protocols are important, but might be trimmed in favor of staying focused on the genetic narrative.

Answer: Thank you for your valuable suggestions. We have streamlined the descriptions of micro-TESE techniques and IVF-ICSI protocols to focus more clearly on the genetic narrative. Page 3-4, line 69-84.

-Redundant Phrasing: Some information is repeated (e.g., the diagnostic limitations of current methods); a tighter structure can avoid diluting your key message.

Answer: Thank you for your valuable suggestions. We have modified the relevant information in the revised version. Page 3-4, line 69-84.

-Spermatogenesis Stages: Briefly mention the complexity without breaking down every sub-stage unless it's directly relevant to gene-pathology links.

Answer: Thank you for your valuable suggestions. We have modified the relevant information in the revised version. Page 3-4, line 69-84.

-I'd appreciate even just 3–5 well-established genes associated with NOA (e.g., TEX11, NR5A1, SYCP3, DMRT1)

Answer: Thank you for your valuable suggestions. We have added the relevant information in the revised version. Page 4, line 87-88.

**Results**

ome minor points:

“Publication bias were…” → should be “was.”

Answer: Thank you for your detailed suggestions. We have revised them in the updated version. Page 7, line 164, line 173.

“TEST outcomes” likely meant “TESE outcomes.”

Answer: Thank you for your detailed suggestions. We have revised them in the updated version. Page 8, line 179.

“Include study” should be “included study.”

Answer: Thank you for your detailed suggestions. We have revised them in the updated version. Page 7, line 168.

Main Concern:

-The diagnostic yield of 15% with high heterogeneity (I² = 90%) weakens the generalizability of the findings. Even after excluding the outlier study, the I² drops to 79%, which still reflects substantial variability.

Recommendation: Clarify sources of heterogeneity—e.g., were there differences in ES platforms, inclusion criteria, or variant interpretation pipelines? A subgroup or meta-regression analysis would strengthen your conclusions.

Answer: We appreciate your valuable suggestions and have addressed the potential sources of heterogeneity by providing a clearer discussion in the revised manuscript. Page 8-9, line 198-206.

Due to the limited number of included studies and small sample size of these studies, we did not perform subgroup analysis or meta-regression; we acknowledge this as a limitation.

-The initial funnel plot bias is statistically non-significant (P=0.46), which is good—but after exclusion, bias becomes significant (P=0.01). That’s a red flag.

Suggestion: Provide a rationale for why publication bias increased after refining your dataset. Was the excluded study larger/smaller or methodologically distinct?

Answer: We appreciate your detailed suggestions. In the revised manuscript, we have discussed the increased risk of publication bias following the exclusion of the study with the largest sample size. This may be attributed to the "small-study effect," whereby smaller studies are often conducted in specialized centers that selectively enroll patients with more severe or familial phenotypes, potentially leading to an inflated diagnostic yield. Page 8, line 206-209.

-Mentioning 264 genes across 270 cases underscores the extreme genetic heterogeneity of NOA. However, listing only the top eight without describing their functions or known pathogenicity misses an opportunity.

Stronger framing would include:

1.Whether these gene variants were classified as pathogenic/likely pathogenic or VUS.

2.Functional categories (e.g., meiosis-related, DNA repair)

3.Recurrence rates—were any genes found in independent cohorts?

The phenotype-genotype analysis is a great addition. Highlighting positive sperm retrieval rates by histopathological subtype adds clinical relevance.

Still, the n is very low for retrieval outcomes (only 45 cases with TESE info). Drawing broad predictive conclusions from this is risky.

Recommendation: Be more cautious in your language; frame these associations as preliminary observations rather than definitive predictors.

Answer: We gratefully acknowledge the reviewer's insightful suggestions and have fully incorporated the feedback by revising the discussion section in accordance with the recommendations. Page 9-10, line 222-229; Page 10, line 234-237.

**Discussion**

1. Structure and Flow

The discussion would benefit from clearer paragraph divisions. Right now, multiple ideas are packed into long blocks of text. Suggest splitting into sections like:

-Diagnostic yield and heterogeneity

-Genetic insights and gene-specific findings

-Clinical correlation with TESE outcomes

-Broader implications and future directions

Answer: We gratefully acknowledge your valuable suggestions and have accordingly divided the discussion section as recommended. Page 8-12.

2. Overuse of Numbers Without Interpretation

While numbers like “264 genes in 270 cases” or “2,274 testis-expressed genes” are impressive, they need context. What proportion are known monogenic causes? How many are novel?

Answer: We appreciate your thoughtful inquiry and have incorporated the relevant information in the revised manuscript to enhance clarity and completeness. Page 10, line 231-234.

3. Insufficient Functional Context for Key Genes

AR, TEX11, FANCM, etc., are cited as “top genes,” but their biological roles and clinical significance are not explained. Including 1–2 lines describing their mechanistic relevance would add depth.

Answer: We gratefully acknowledge your insightful suggestions. In accordance with your recommendation, we have incorporated a detailed discussion of the biological functions, clinical implications, and mechanistic relevance of the "top genes" in the Discussion section. Page 10, line 237-246.

4. TESE Outcomes: Careful Framing

The 11.11% TESE success rate in ES-positive cases is a striking finding, but be cautious in interpretation:

Small sample size (only 45 reported cases)

Possibly skewed by selection bias

Could reflect enrichment for more severe phenotypes

Frame it as hypothesis-generating, not definitive.

Answer: Thank you for your valuable suggestions. In accordance with your recommendation, we have reinterpreted the TESE success rate observed in this meta-analysis, which is based on a small sample size. Page 11, line 263-266.

5. AI Model Mention

The mention of an AI model is promising but abrupt. If it's important, integrate it earlier with more detail or drop it for cohesion.

Answer: We appreciate your valuable suggestion and have removed the description regarding the AI model to maintain overall coherence. Page 12.

**Reviewer #2: The authors have written an important and timely meta-analysis addressing the diagnostic yield of exome sequencing (ES) in nonobstructive azoospermia (NOA) with rigorous methodology. However, there are several areas that require clarification and improvement:**

1. GRADE Assessment Not Reported: While the methods mention that the certainty of evidence was assessed using GRADE criteria, the results of this assessment are not shared anywhere in the manuscript. Please include a summary of the GRADE findings, either in the main text or as a supplementary table.

Answer: We gratefully acknowledge your valuable suggestions. A summary of the GRADE findings has been included in Supplementary File 2. Page 7-8, line 174-176.

2. Sensitivity Analysis Missing: Sensitivity analysis is described in the methodology section, but no results from such an analysis are presented in the results or supplementary material. Kindly clarify whether this was performed and, if so, summarize the findings.

Answer: We gratefully acknowledge your insightful suggestions. The sensitivity analysis, conducted by sequentially excluding individual studies, has been summarized and presented in Supplementary File 2. Page 8, line 174-176.

3. Limited Exploration of Predictive Value for TESE: The study rightly mentions the limited predictive value of ES for testicular sperm extraction (TESE) outcomes, but this point is not explored in sufficient depth. A more nuanced discussion on how specific gene variants or histopathological phenotypes correlate with TESE success would enhance the clinical relevance of the findings.

Answer: We gratefully acknowledge your valuable suggestions. In response, we have incorporated a more comprehensive discussion on the associations between specific gene variants or histopathological phenotypes and TESE outcomes, informed by current evidence. We further emphasize that the relationship between histopathological characteristics and underlying genetic etiologies in individuals with NOA remains an active area of investigation. Page 11-12, line 268-282.

4. Heterogeneity Not Addressed in Discussion: The meta-analysis demonstrates substantial heterogeneity (I² = 90%), yet this is not adequately addressed in the discussion section. Please elaborate on potential sources of heterogeneity (e.g., study design, population differences, sequencing methods, variant interpretation criteria) and their impact on the robustness of your conclusions.

Answer: We gratefully acknowledge your insightful suggestions. In the revised manuscript, we have thoroughly examined the potential sources of heterogeneity and carefully evaluated their implications for the robustness of our conclusions. Page 8-9, line 198-216.

Addressing these points will significantly improve the clarity, rigor, and impact of your manuscript.
